# Medical Service in the Navy

**Published:** 1859-01

**Authors:** 


### *Medical Service in the Navy.*

The Hon. Isaac Toucey, Secretary of the Navy, in his Annual Report for 1858, states that the present effective strength of the medical corps of the navy does not exceed 59 surgeons and 70 assistant surgeons. "The wants of the service for the ensuing year [1859] will require for duty at sea, 39 surgeons and 70 assistants; for shore duty 26 surgeons and 17 assistants; making a total of 65 surgeons and 87 assistants, required for active duty. This deficiency in the medical corps of the navy cannot be neglected without subjecting the lives of those engaged in this branch of the public service to unreasonable and culpable exposure."

The aggregate number of medical officers required for active duty is 152; but the aggregate number now in the navy is 149, and of these at least 20 are incapacitated for active service.

In the year 1815, the aggregate number of medical officers was 129. Since that time the number of captains has been increased from 32 to 76; of commanders from 18 to 106; of lieutenants from 140 to 327; but the medical corps has been increased from 120 only to 149.

For these reasons, among others, the Secretary recommends Congress to make an addition of 20 surgeons and 20 assistants to the medical corps of the navy. "With that number the stations on shore and vessels at sea may be supplied, and a short interval of relief from duty allowed an officer on his return from a cruise."

To supply the deficiencies arising from deaths and resignations, the appointments have been from 6 to 8 yearly. But if Congress should increase the medical corps in accordance with the recommendation of the Secretary of the Navy, it is probable that about fifty young gentlemen will be appointed to meet the demands of the service during the year 1859. Candidates for admission into medical corps of the navy will not often find a more favorable oppportunity. It is presumable, therefore, that a considerable number of the students now preparing in the several colleges of this and other cities, to graduate next spring, will apply to the Secretary of the Navy for permission to be examined by the Board of Naval Surgeons, which is usually convened about the close of the lecture season, to select persons suitable for the office of assistant surgeon in the navy. It is understood that the Board selects the number required for the year, from those examined. The places are put up for competition, and are given to those best qualified in all respects; but no one can incur the odium that is supposed to attach to an absolute rejection, though he may feel a little chagrined to find that his competitors have been more successful than himself.

The Government seeks to employ in the Medical department of the army and navy only those who are properly qualified to practice medicine and surgery advantageously to the thousands of our fellow citizens, soldiers and sailors, officers and privates, engaged in its military service, whose lives are frequently contingent upon the professional knowledge and judgement of their medical advisers. Without the chance of consultation with experienced practitioners, the assistant surgeon may be required, immediately after appointment, to operate, to dress wounds, and prescribe for the sick. Responsibilities, anxieties and labor belong to the position.

The qualifications necessary to fill the office in the navy are vigorous health, a sound condition of all the senses, an orderly, quick intellect, and integrity of moral character. The age of the candidate must not exceed twenty-five years.

The young medical officer is required to keep a professional journal, a register of the names, etc., of patients treated, a record of expenses incurred on their account, and to prepare from these periodically, reports in tabular form, for the information of the government. For this reason, it is very desirable that he should be able to write English correctly, legibly and readily, and understand very accurately the primary rules of arithmetic and the statement of ordinary accounts.

As he is often under the necessity of putting up his own prescriptions, he is expected to be familiar with the physical properties of drugs in common use, and the methods of weighing, measuring and compounding them accurately, as well as with the modes of preparing articles of diet for the sick. Indeed, the young officer will speedily discover on board of a ship of war that he has more frequent occasion to exercise his knowledge of materia medica, pharmacy and therapeutics—to use measure-glass, scales, pestle and mortar, and spatula—

4

than the trephine or amputating knife. The necessity for exstracting teeth, or the abstraction of blood by lancet or cups, or the preparation of cataplasms, daily occurs. Such details of practice as are left, especially in cities, to experienced nurses or to others, demand on board of ship, the supervision, at least, of the medical officer himself; and therefore it is peculiarly proper that he should be acquainted with these seemingly little things, on assuming the duties of his office.

Candidates are expected to evince a fair knowledge of the elementary and general principles of chemistry, of physiology, of general and surgical anatomy, of medical jurisprudence, of the principles and practice of surgery and medicine, and of obstetrics, as taught in the schools. Upon all these points, the examinations are minute, and as far as possible, practical. The candidate is afforded opportunities to distinguish drugs, to compound prescriptions, to diagnose diseases at the bedside, to apply surgical dressings and apparatus, and to operate on the cadaver in the presence of the examiner: and, as the examination is partly by writing, partly oral and partly practical, he is thus enabled to exhibit fairly to what degree he is prepared to enter immediately on the active duties of the profession.

All the qualifications which are befitting to a Christian gentleman in any condition of life, are becoming to the medical officer of the army or navy. Collateral sciences and the languages, modern and ancient, *are not essential* to successful competition for the office of assistant surgeon in the navy; but as the possession of this kind of knowledge is indicative, to some degree at least, of industry and mental habit and capacity, they are considered in the classification of those selected for appointment—the most accomplished in the estimation of the Board, being placed highest on the list. But without an adequate knowledge of the principles of the profession, and aptitude in their application, mere accomplishments in the physician, are regarded to be of little value.—*Medical & Surgical Reporter.*

